# mTOR-dependent dysregulation of autophagy contributes to the retinal ganglion cell loss in streptozotocin-induced diabetic retinopathy

**DOI:** 10.1186/s12964-020-00698-4

**Published:** 2021-02-26

**Authors:** Sanjar Batirovich Madrakhimov, Jin Young Yang, Jin Ha Kim, Jung Woo Han, Tae Kwann Park

**Affiliations:** 1 Department of Interdisciplinary Program in Biomedical Science, Soonchunhyang Graduate School, Bucheon Hospital, Bucheon, South Korea; 2 Laboratory for Translational Research On Retinal and Macular Degeneration, Soonchunhyang University Hospital Bucheon, Bucheon, South Korea; 3 Department of Ophthalmology, Soonchunhyang University Hospital Bucheon, Bucheon, South Korea; 4 Department of Ophthalmology, College of Medicine, Soonchunhyang University, Choongchungnam-do, Cheonan, South Korea; 5 Department of Ophthalmology, College of Medicine, Soonchunhyang University Bucheon Hospital, Bucheon, South Korea; 6 Ex Lumina Therapeutics and Technologies. Co., Ltd., Bucheon, South Korea

**Keywords:** Diabetic retinopathy, Neurodegeneration, Autophagy, Apoptosis, The mechanistic target of rapamycin (mTOR)

## Abstract

**Background:**

Neurodegeneration, an early event in the pathogenesis of diabetic retinopathy (DR), precedes clinically detectable microvascular damage. Autophagy dysregulation is considered a potential cause of neuronal cell loss, however underlying mechanisms remain unclear. The mechanistic target of rapamycin (mTOR) integrates diverse environmental signals to coordinate biological processes, including autophagy. Here, we investigated the role of mTOR signaling in neuronal cell death in DR.

**Methods:**

Diabetes was induced by a single intraperitoneal injection of streptozotocin and tissue samples were harvested at 1, 2, 3, 4, and 6 months of diabetes. Early-stage of DR was investigated in 1-month-diabetic mice treated with phlorizin (two daily subcutaneous injections at a dose of 200 mg/kg of body weight during the last 7 full days of the experiment and the morning of the 8th day, 3 h before sacrifice) or rapamycin (daily intraperitoneal injections, at a dose of 3 mg/kg for the same period as for phlorizin treatment). The effect of autophagy modulation on retinal ganglion cells was investigated in 3-months-diabetic mice treated with phlorizin (two daily subcutaneous injections during the last 10 full days of the experiment and the morning of the 11th day, 3 h before sacrifice) or MHY1485 (daily i.p. injections, at a dose of 10 mg/kg for the same period as for phlorizin treatment). Tissue samples obtained from treated/untreated diabetic mice and age-matched controls were used for Western blot and histologic analysis.

**Results:**

mTOR-related proteins and glucose transporter 1 (GLUT1) was upregulated at 1 month and downregulated in the following period up to 6 months. Diabetes-induced neurodegeneration was characterized by an increase of apoptotic marker—cleaved caspase 3, a decrease of the total number of cells, and NeuN immunoreactivity in the ganglion cell layer, as well as an increase of autophagic protein. Insulin-independent glycemic control restored the mTOR pathway activity and GLUT1 expression, along with a decrease of autophagic and apoptotic proteins in 3-months-diabetic mice neuroretina. However, blockade of autophagy using MHY1485 resulted in a more protective effect on ganglion cells compared with phlorizin treatment.

**Conclusion:**

Collectively, our study describes the mechanisms of neurodegeneration through the hyperglycemia/ mTOR/ autophagy/ apoptosis pathway.

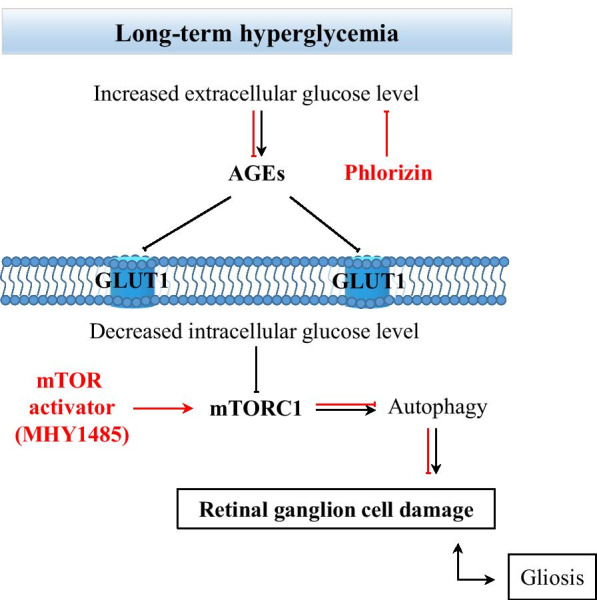

**Video Abstract**

**Supplementary Information:**

The online version contains supplementary material available at 10.1186/s12964-020-00698-4.

## Background

Diabetic retinopathy (DR) is a severe ocular complication of diabetes mellitus (DM) leading to vision loss in adults aged 20–79 years [1, 2]. The prevalence of DR ranges from 21.7 to 34.6% among diabetic individuals [2–5], whose number was estimated at 451 million of adults in 2017 and is projected to increase to 693 million worldwide by 2045 [6]. Despite the introduction of various screening programs for DR detection, it remains the leading cause of blindness for 2.6 million adults among the global population [7, 8].

DR is a multifactorial disease with extremely complex mechanisms of development [9]. DR is classified into non-proliferative diabetic retinopathy and proliferative diabetic retinopathy [10, 11]. These “early” and “advanced” stages are characterized by the level of microvascular and ischemic damage of the retina, which determines visible clinical manifestations [11]. However, advances in retinal imaging techniques and electrophysiological studies demonstrated subtle functional deficits and neuronal abnormalities in patients without background retinopathy [12–19]. Experimentally, aside from compromise of vascular cells, alterations of various retinal neuronal cells, macroglia and microglia have been shown in diabetes [20–27]. Thus, the classic view on DR as the microcirculatory pathology evolved into the concept of disease of the retinal neurovascular unit (NVU), where neurodegeneration precedes clinically detectable microvascular damage [11, 28–31].

**Fig. 1:**
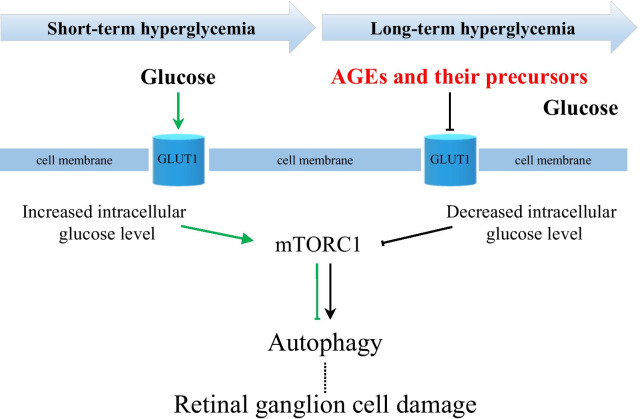
Schematic of the hypothesis. Schematic representation of mTORC1 activity regarding the duration of hyperglycemia and its consequences for retinal ganglion cells. AGEs—advanced glycation end-products

The concept of retinal NVU describes neurons, glial, and vascular cells as a functional and structural integration, which is essential for maintaining the homeostasis in the inner retina [11, 28–30, 32]. Under diabetic conditions, such as hyperglycemia, abnormal blood flow, and others, the NVU appears to be an adaptive mechanism, which fails after prolonged metabolic perturbations [29, 31]. All components of the NVU are affected by diabetes, but which cells are primarily involved in pathologic processes remain unclear [32, 33]. Although vascular, glial, and neuronal cell damage appears to be interdependent, the latter significantly contributes to the development of DR by triggering molecular events resulting in the blood-retinal-barrier dysfunction, microangiopathy, and the proinflammatory milieu [28, 31, 33, 34]. One of the hallmarks of neuronal cell damage is persistent apoptosis, which may be initiated by several pathways, such as glutamate excitotoxicity, loss of neuroprotective factors, oxidative stress, and neuroinflammation [31, 34, 35]. However, growing evidence suggests that defective autophagy also contributes to the diabetes-induced neuronal cell death [36–39]. Autophagy is an adaptive mechanism that may be upregulated against various stress conditions, such as nutrient deprivation, oxidative stress, and others to maintain cellular homeostasis by lysosomal degradation of damaged intracellular elements, and supplying with nutrients [40–42]. However, dysregulated autophagy may have fatal consequences for the cell, indicating a complex network between autophagy and apoptosis [40–42]. At this point, we wondered that if autophagy dysregulation involved in diabetes-induced neurodegeneration, what is the role of the mechanistic target of rapamycin (mTOR) pathway in this context, which is the master regulator of autophagy. This was a starting point of our investigation and we hypothesized how the mTOR pathway may contribute to the neuronal cell death in the DR (Fig. 1). To address the question, we studied the dynamics of events leading to the neuronal cell death in a streptozotocin(STZ)-induced mouse model of diabetes over 6 months of period. The results demonstrated that activity of the mTOR pathway was initially increased at early period of diabetes and this increase was reversed by insulin-independent glycemic control. At advanced stages of diabetes, we observed downregulation of the mTOR activity accompanying with autophagy activation and neuronal cell loss. Furthermore, the management of hyperglycemia could restore the mTOR activity, inhibit autophagy and prevent neuronal cell death. Interestingly, treatment with the mTOR activator, which is MHY1485, demonstrated a more prominent protective effect on neuronal cells even under hyperglycemic conditions. Therefore, downregulation of mTOR activity by various factors associated with prolonged hyperglycemia may be responsible for the autophagy-induced neuronal cell death in the DR. These findings have implications for the development of mTOR-based therapies to manage autophagy-related neuronal cell death in the early stages of DR.

## Materials and methods

### Animal care

All animal experiment was designed and conducted in accordance with the Guide of the Care and Use of Laboratory Animals, the Association for Research in Vision and Ophthalmology Statement for the Use of Animals in Ophthalmic and Vision Research, and approved by the Institutional Animal Care and Use Committee for Soonchunhyang University Hospital Bucheon.

Mice used in this study were C57/BL6 strain (8 weeks, male, 22–24 g) and purchased from the Orient Bio Inc., (Seongnam, South Korea). All mice were housed in breeding cages in a room with a 12/12-h light/dark cycle and had free access to food and water. The humidity and temperature were respectively maintained at 50% and 23–26 °C.

### Induction of diabetes

Diabetes was induced by a single i.p. injection of STZ (150 mg/kg; Sigma-Aldrich, St. Louis, MO, USA). STZ was freshly prepared in 100 mM citrate buffer (pH 4.5). To avoid sudden hypoglycemia post-injection, the mice were fed 10% sucrose overnight. Hyperglycemia was confirmed by measuring blood glucose using the Accu-Check active blood glucose monitor (Roche Diagnostics, Mannheim, Germany) 2 days and 2 weeks post-injection. Only mice with non-fasting blood glucose levels > 300 mg/dL, polyuria, or glucosuria were defined as STZ-induced diabetic mice and used in the experiments. Non-diabetic, age-matched mice were used as the controls. The general parameters of mice obtained on the day of the sacrifice are given in Table 1.

**Table 1:** Body weight and blood glucose level dynamics

Group	n	Body weight (gr)	Glucose level (mg/dL)	Number of mice used for each	
		Mean ± SD	Mean ± SD	WB	IF
*a*					
Normal	10	30.5 ± 1.2	161.9 ± 14.1	6	4
1 m DM	10	21.2 ± 0.8	448 ± 51.3	6	4
2 m DM	10	24.8 ± 1.2	458.7 ± 67	6	4
3 m DM	10	25.7 ± 1.7	467.8 ± 72.3	6	4
4 m DM	9	28.5 ± 2	500.5 ± 71.1	6	3
6 m DM	8	29 ± 1.1	481.5 ± 65.9	5	3
*b*					
Normal	10	22.25 ± 0.8	165.5 ± 20.9	6	4
1 m DM	10	21.05 ± 0.7	488.6 ± 63.8	6	4
1 m DM/PHL	10	21.4 ± 1.7	231.3 ± 43.9	6	4
1 m DM/Rapa	9	20.85 ± 2.4	461.2 ± 53.4	6	3
*c*					
Normal	10	27.55 ± 0.9	163 ± 14.7	6	4
3 m DM	9	25.6 ± 1.5	466.7 ± 71.8	6	3
3 m DM/PHL	9	25.9 ± 1.6	198 ± 38.9	6	3
3 m DM/MHY	8	24.8 ± 1.2	470.7 ± 73.1	5	3

### Grouping and treatment regimen

**Fig. 2:**
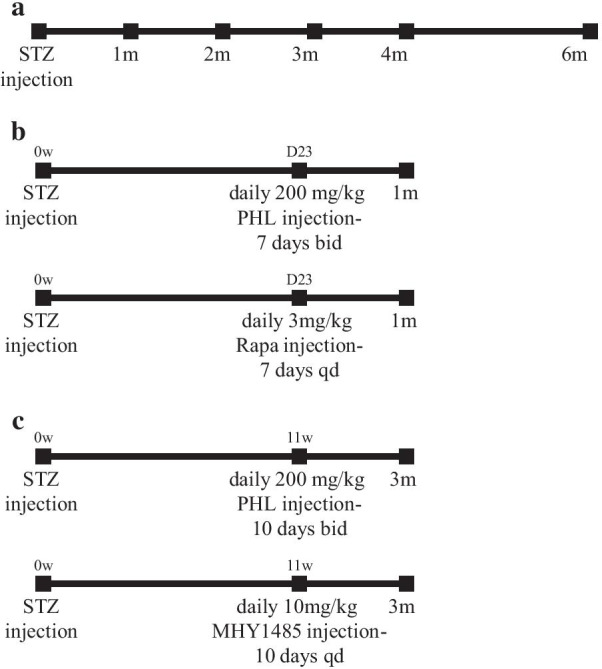
Study design and general parameters of mice; bid—twice a day, qd—once a day. Table 1 Body weight and blood glucose level dynamics

Total of 140 diabetic animals was used in this study. To evaluate the natural course of DR, we observed 60 mice over 6 months of period. Animals were assigned to 6 groups depending on observation period: (1) Normal control, (2) 1 m DM, (3) 2 m DM, (4) 3 m DM, (5) 4 m DM and (6) 6 m DM (Fig. 2a). The effects of short-term hyperglycemia on the retina were investigated on 40 mice, divided into 4 groups: (1) Normal control, (2) 1 m DM, (3) 1 m DM/PHL (4) 1 m DM/Rapa (Fig. 2b). Mice in the 1 m DM/PHL group received two daily subcutaneous injections of phlorizin (PHL) (Cayman Chemical, Ann Arbor, MI) dissolved in 60% propylene glycol in phosphate-buffered saline at a dose of 200 mg/kg of body weight during the last 7 full days of the experiment and the morning of the 8th day, 3 h before sacrifice. PHL, a phloretin glucoside, restores blood glucose level to the normal by increasing glucose excretion by kidney [43]. The dosage and the way of administration of PHL was chosen based on previous studies [44, 45]. Mice in the 1 m DM/Rapa group, received daily i.p. injections of rapamycin (Sigma-Aldrich, St. Louis, MO) diluted in 4% ethanol and 5% Tween-20 in distilled water at a dose of 3 mg/kg for the same period as for the 1 m DM/PHL group [46]. Rapamycin, a macrolide, inhibits mTORC1 after forming a gain-of-function complex with FKBP12 protein [47].

The effects of long-term hyperglycemia on the retina of diabetic mice were evaluated on 40 mice, divided into 4 groups: (1) Normal control, (2) 3 m DM, (3) 3 m DM/PHL, and (4) 3 m DM/MHY (Fig. 2c). Mice in the 3 m DM/PHL group received two daily subcutaneous injections of PHL during the last 10 full days of the experiment and the morning of the 11th day, 3 h before sacrifice. Mice in the 3mDM/MHY group received daily i.p. injections of MHY1485 (MedChem Express, Monmouth Junction, NJ) at a dose of 10 mg/kg for the same period as for the 3 m DM/PHL group. MHY1485, an mTOR activator, inhibits the fusion between autophagosomes and lysosomes, thus downregulates autophagy [48, 49]. Eight diabetic mice died in the course of this study.

### Tissue collection and preparation

After completing the experiment, mice were used for WB and histologic analysis. For histologic analysis, mice were deeply anesthetized by intraperitoneal injection using the mixture of 40 mg/kg zolazepam/tiletamine (Zoletil 50, Virbac, Carros Cedex, France) and 5 mg/kg xylazine (Rompun, Bayer Healthcare, Leverkusen, Germany) and perfused intracardially with 0.1 M phosphate buffer (PB) containing 1000 U/ml of heparin, followed by an infusion of 4% paraformaldehyde (PFA) in 0.1 M PB. The eyecup was made by removing the anterior segment from the enucleated mouse eye and fixed with 4% paraformaldehyde (Biosesang, Seongnam, South Korea) for 1 h, dehydrated in 30% sucrose overnight, and embedded in frozen section compound (Leica Biosystems Richmond, IL). The sample collection for the WB experiment was that the neuroretina was separated from the enucleated mouse eyes, pooled together and stored at − 80 °C until the experiment.

### Western blot

Neuroretinal tissue was disrupted in RIPA II lysis buffer (Gendepot, Barker, Tx) containing Xpert phosphatase inhibitor cocktail (Gendepot, Barker, Tx) and Xpert protease inhibitor cocktail (Gendepot, Barker, Tx) for 1 h on the ice. Insoluble material was removed by centrifugation (13,000 rpm, 15 min, 4 °C), and the only supernatant was obtained. Protein lysate was quantified using Pierce BCA Protein Assay kit (Thermo scientific, Middlesex, MA) according to the manufacturer’s instruction.

The lysates were denatured in 4 × Laemmli sample buffer (Gendepot, Barker, Tx), boiled for 10 min at 95 °C. Aliquots of each sample with an equal amount of protein were separated using SDS-acrylamide gel in the range from 6 to 10%, depending on the molecular weight of the target antibody and transferred onto polyvinylidene fluoride membrane (ATTO, Amherst, NY). The membranes were incubated with 5% skim milk in Phosphate-Buffered Saline (PBS) containing 0.1% Tween-20 for detection of total proteins and 5% BSA in PBS containing 0.1% Tween-20 for phosphorylated proteins for 1 h at room temperature and incubated with anti-mTOR, anti-phospho-S6 ribosomal (pS6) protein (S240/244) from Cell signaling technology (Danvers, MA), anti-phospho-mTOR (S2448), anti-GLUT1 from Abcam (Cambridge, UK), anti-β-actin from Santa Cruz Biotechnology (Dallas, TX) antibodies overnight at 4 °C; details on antibodies are given in Additional file 1: Table S1. After washing, the membranes were incubated with horseradish peroxidase (HRP)-conjugated goat anti-rabbit IgG and goat anti-mouse IgG (Genedepot, Barker, Tx) at room temperature for 2 h. The immunoreactive signal was developed using Western blotting detection kit (EzWestLumi plus, ATTO Corporation, Tokyo, Japan) and read with Azure BiosystemsTM c280 (Azure Biosystems, Dublin, CA, USA). Bands on blots were quantified using the ImageJ software (National Institutes of Health, Bethesda, MD).

### Immunofluorescence

Fresh eyecup cryosections (10 μm in thickness) were made with a Cryotome (Thermo Fisher Scientific Shandon Cryotome, Middlesex, MA). The slides were washed with 1xPBS containing 0.1% Triton X-100 (Sigma-Aldrich, St. Louis, MO) and blocked with 5% donkey serum in PBST for 1 h. The blocked slides were then incubated with anti-phospho-mTOR (S2448), anti-GLUT1 from Abcam (Cambridge, UK), anti-phospho-S6 ribosomal protein (S240/244) and anti- Glial fibrillary acidic protein (GFAP) (CST) from Cell signaling technology (Danvers, MA), anti-Glutamine synthetase (GS) from Millipore (Billerica, MA), anti brain-specific homeobox/POU domain protein 3A (Brn3a) and anti-neuronal nuclear protein (NeuN) from Millipore (Billerica, MA), anti Calbindin (Calb) from Sigma-Aldrich (St. Louis, MO), anti-Cleaved caspase-3 from Cell signaling technology (Danvers, MA), anti-Beclin1 from Novus Biologicals (Littleton, CO) antibodies for 2 h and detected with secondary antibodies (Alexa Fluor 488 or 568 or 647, dilution 1:1000; Invitrogen Corp., Carlsbad, CA); details on antibodies are given in Additional file 1: Table S1. Nuclear counterstaining was performed with Hoechst 33,342 (Invitrogen Corp, Carlsbad, CA). After washing, the sections were mounted with fluorescence mounting medium (Dako, Santa Clara, CA).

### Image analysis and cell counting

All slides were imaged using a Leica SP8 (Leica Microsystems, Wetzlar, Germany) confocal microscope. Cleaved caspase-3, NeuN and Hoechst positive cells (n = 6 per group) were manually calculated using Image J software (National Institutes of Health, Bethesda, MD).

### STRING analysis

The potential interactions among selected proteins were analyzed using free online database of currently known proteins—STRING (Search Tool for the Retrieval of Interacting Genes, v11.0). IDs of selected proteins were used as an input in the database (http://string-db.org/) to find functional associations in homo sapiens and mus musculus with a minimum interaction score of 0.400 (medium confidence). Active interaction sources included text mining, experiments, databases, gene fusion, co-occurrence, co-expression, and neighborhood.

### Statistical analysis

Data are expressed as means ± standard deviation. Differences among groups were analyzed using the Mann–Whitney U test. Differences were considered statistically significant when *p* ≤ 0.05.

## Results

### Hyperglycemia-induced molecular events and consequences in STZ mouse retina

**Fig. 3:**
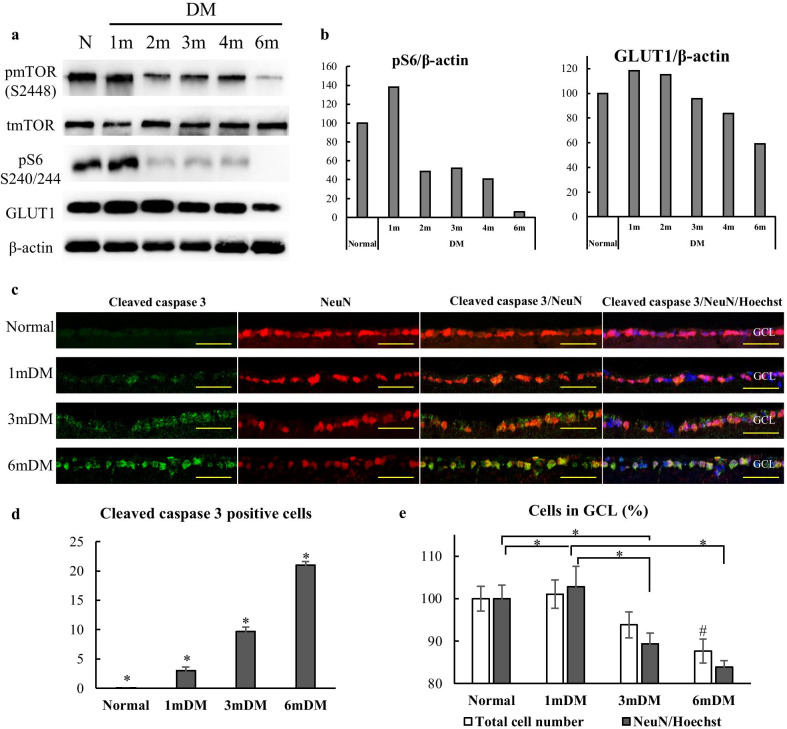
Hyperglycemia-induced molecular events and consequences in STZ mice retina. **a** The expression of mTOR related proteins, GLUT1 and β-actin detected by Western blot. **b** Relative expression of the pS6 ribosomal protein and GLUT1 normalized to β-actin. **c** Cryosections of the mouse retina immunostained for cleaved caspase 3 (green) and NeuN (red), GCL—ganglion cell layer; Scale bar: 50 µm. **d** Cleaved caspase 3 positive cell number in the GCL of the normal and diabetic mouse retina (n = 6/group). **e** Total cell number and NeuN positive cell number (normalized to the Hoechst nuclear counterstain) in the GCL of the normal and diabetic mouse retina. (n = 6/group). ^#^*p* ≤ 0.05, compared with the Normal and 1mDM group; *p ≤ 0.05

We observed the effect of hyperglycemia on the expression of mTOR related proteins and GLUT1 for 6 months after induction of diabetes. After 1 month of STZ injection, pmTOR (S2448) and its downstream effector—pS6 (S240/244) levels were upregulated followed by substantial decrease at 2, 3 and 4 months. Six months samples demonstrated the lowest level of pmTOR (S2448) and pS6 expression (Fig. 3a, b). GLUT1 also showed higher levels at one month after diabetes induction, and then gradually decreased by 6 months of observation (Fig. 3a, b).

To evaluate neuronal cell death in the diabetic retina, we immunostained the cryosections of the retina of STZ mice at 1, 3, 6 months for Cleaved caspase-3 and NeuN, normal mouse retina served as control (Fig. 3c). Cleaved caspase-3 signals were detected in the ganglion cell layer (GCL) of the diabetic retina, which was weak at 1 month and strong at 3 and 6 months (Fig. 3c), positive signal was not observed in normal retina. Counting of Cleaved caspase-3-positive cells demonstrated a gradual increase of apoptotic cell number and a peak after 6 months of diabetes induction (*p* < 0.05, Fig. 3d). In addition, NeuN immunoreactivity of ganglion cells progressively decreased at 3 and 6 months, which was also confirmed by counting of NeuN-positive cells (*p* < 0.05) (Fig. 3c, e). The total number of cells in GCL was also decreased after 3 (*p* > 0.05) and 6 months of diabetes (*p* < 0.05) (Fig. 3e).

### pmTOR S2448, pS6 and GLUT1 expressing cells in the normal mouse retina

**Fig. 4:**
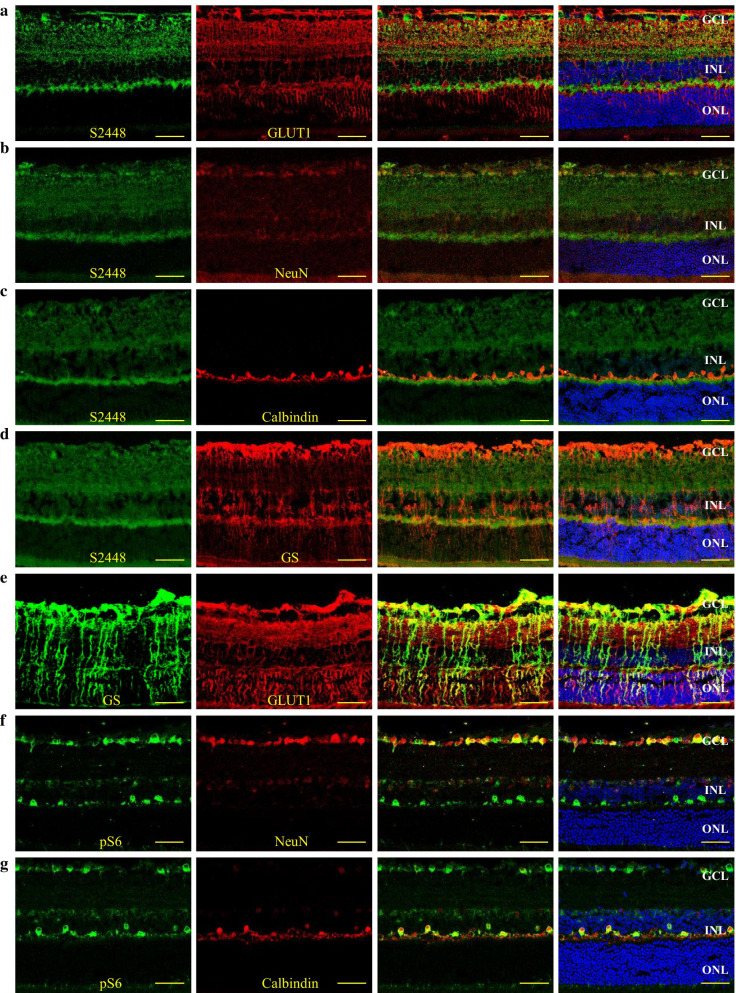
pmTOR S2448, pS6 and GLUT1 expressing cells in the normal mouse retina. Cryosections of normal mouse immuno-co-stained with *pmTOR S2448* (green) and *GLUT1* (red) (**a**), or *NeuN* (red) (**b**), or *Calbindin* (red) (**c**), or *Glutamine synthetase* (GS—red) (**d**), *GS* (green) and *GLUT1* (red) (**e**), *pS6* (green) and *NeuN* (red) (**f**), or *Calbindin* (red) (**g**). Nuclei were counter-stained with *Hoechst 33,342* (blue). GCL, ganglion cell layer; INL, inner nuclear layer; ONL, outer nuclear layer; *Scale bar: 50 µm*

Next, we performed immunofluorescence assay to identify pmTOR S2448, pS6 and GLUT1 expressing cells in the neuroretina of normal mouse. pmTOR S2448 is expressed in the GCL, in both plexiform layers and the cell bodies in the inner nuclear layer (INL), as was found to be the similar for GLUT1, which was also expressed in the cell bodies of the outer nuclear layer (ONL) (Fig. 4a). Co-immunostaining of pmTOR S2448 and NeuN confirmed the expression of activated mTORC1 in ganglion cells (Fig. 4b). Phosphorylated mTOR at S2448 was also expressed in Calbindin positive cells (Fig. 4c). Moreover, pmTOR S2448 is expressed in the endfeet and cell bodies of Müller cells immunostained for Glutamine synthetase (GS, Fig. 4d), which was also co-localized with GLUT1 (Fig. 4e).

The downstream effector of mTORC1 complex, pS6, was found to be expressed highly in the GCL and the outermost part of the INL, weakly in the cell bodies in the inner half of the INL. While the source of the pS6 signal in the GCL was NeuN-positive ganglion cells (Fig. 4f), signals from the outermost part of the INL originated from the cell bodies and processes of horizontal cells immunostained for Calbindin (Fig. 4g). Weak pS6 signal colocalized with NeuN positive cells in the inner nuclear layer, which are likely to be amacrine cells or ectopic ganglion cells (Fig. 4f).

### Dynamics of hyperglycemia-induced pS6 and GLUT1 expression in STZ mouse retina

**Fig. 5:**
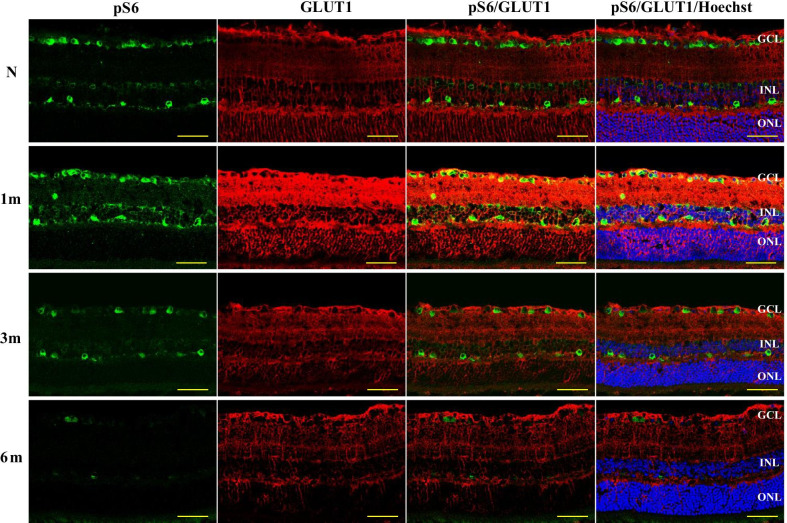
pS6 and GLUT1 expression dynamics over 6 months of observation of STZ-induced DM mice

After 1 month of hyperglycemia, we observed upregulation of pS6 expression in the inner retina. After 3 months of hyperglycemia, pS6 expression was downregulated, amacrine cells and processes of horizontal cells lacked pS6, while cell bodies of ganglion cells and horizontal cells retained pS6 expression. A few pS6-positive ganglion cells and horizontal cells were observed after 6 months of diabetes (Fig. 5). GLUT1 expression was also increased at 1 month of diabetes and was gradually decreased evenly in the neuroretina at 3 and 6 months of diabetes (Fig. 5).

### Hyperglycemia-induced activation of glia and autophagy dysregulation in STZ-mouse retina

**Fig. 6:**
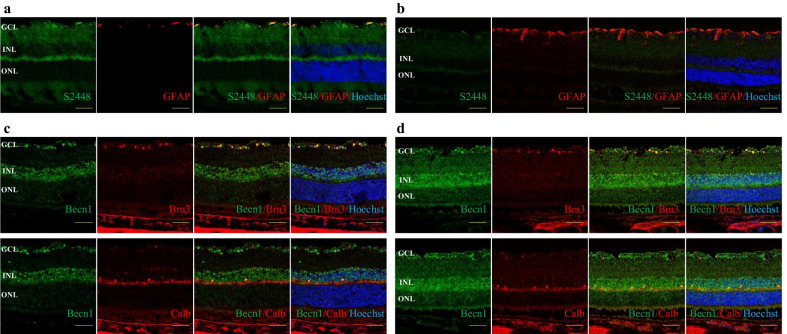
Autophagy and glia activation in the retina of STZ-induced DM. Immuno-co-staining for pmTOR S2448 (green) and GFAP (red) of normal (**a**) and 3 m DM mouse retina (**b**). Immuno-co-staining for Beclin1 (green) and Brn3 or Calbindin (red) of normal (**c**) and 3 m DM mouse retina (**d**). GCL, ganglion cell layer; INL, inner nuclear layer; ONL, outer nuclear layer; Scale bar: 50 µm

Glia reactivity was detected by immunostaining of cryosections of normal and 3 m STZ mouse retina with antibody against glial fibrillary acidic protein (GFAP). In normal mouse retina, GFAP expression was limited to the astrocytes and Müller cell endfeet in the nerve fiber layer (NFL) and GCL (Fig. 6c), whereas in the 3 m DM mouse retina, GFAP expression was elevated and extended to the glia in the NFL, GCL and IPL (Fig. 6d). Moreover, in the 3 m DM mouse retina, pmTOR S2448 expression was evenly downregulated comparing to those of the normal mouse retina (Fig. 6c, d).

To evaluate autophagy flux, we immunostained cryosections of normal and 3 m STZ-mouse retina for Beclin1. In the normal mouse retina, Beclin1 expression which was detected in the GCL, INL and OPL in the normal mouse retina. Beclin1 immunoreactivity in 3 m STZ mouse retina was clearly upregulated and extended to the IPL, ONL and photoreceptor cell layer (Fig. 6a, b).

### The effects of short-term hyperglycemia on STZ-mouse retina are reversible by glycemic control

**Fig. 7:**
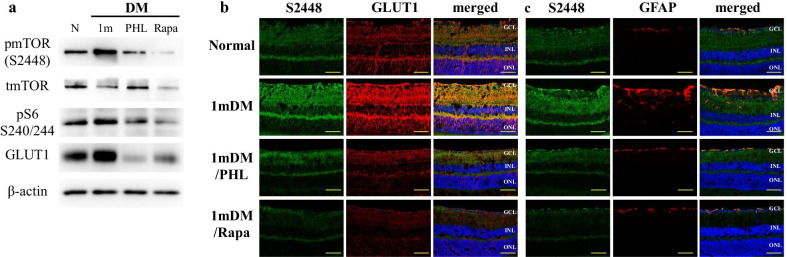
The effects of short-term hyperglycemia on STZ-mouse retina are reversible by glycemic control. **a** The expression of mTOR related proteins, GLUT1 and β-actin detected by Western blot. **b** Immuno-co-staining for *pmTOR S2448* (green) and *GLUT1* (red) of the retina from normal and 1-month diabetic mice treated with either phlorizin or rapamycin. **c** Immuno-co-staining for *pmTOR S2448* (green) and *GFAP* (red) of the retina from normal and 1-month diabetic mice treated with either phlorizin or rapamycin (**a**). Nuclei were counter-stained with *Hoechst 33,342* (blue). GCL, ganglion cell layer; INL, inner nuclear layer; ONL, outer nuclear layer; *Scale bar:* 50 µm

pmTOR S2448 expression was upregulated at 1 month of diabetes and phlorizin (PHL) treatment demonstrated similar levels as those of normal mouse neuroretina demonstrated by Western Blot and immunofluorescence assay. Hyperglycemia-induced upregulation of pS6, as well as GLUT1 levels, were also decreased by glycemic control with PHL treatment. As expected from mTOR inhibition, rapamycin treatment resulted in decreased expression of pmTOR S2448 and pS6 levels, but we also observed downregulation of GLUT1 (Fig. 7a, b).

Glial response was monitored by immunofluorescence assay. In the normal mouse retina, GFAP expression was confined to astrocytes in the NFL and GCL, whereas in the 1 m DM retina, astrocytes located in the inner plexiform layer (IPL) were also stained along with rare Müller glia resembling GFAP positive cells. PHL and rapamycin treatment returned the levels of GFAP expression back to the normal state (Fig. 7c).

### Autophagy dysregulation contributes to neuronal damage after long-term hyperglycemia

**Fig. 8:**
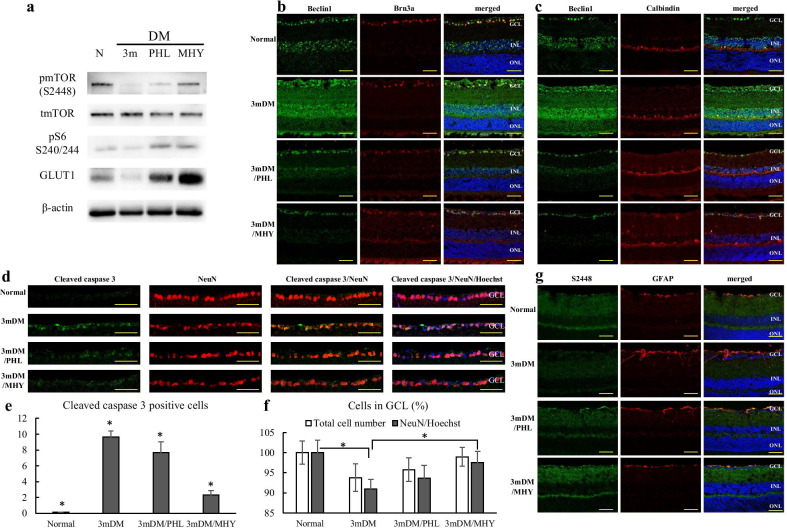
Autophagy dysregulation contributes to neuronal damage after long-term hyperglycemia. **a** The expression of mTOR related proteins, GLUT1 and β-actin detected by Western blot. Immuno-co-staining for Beclin1 (green) and Brn3a (red) (**b**) or Calbindin (red) (**c**) of the retina from normal and 3-months diabetic mice treated with either phlorizin or MHY1485. **d** Immuno-co-staining for Cleaved caspase 3 (green) and NeuN (red) of the retina from normal and 3-months diabetic mice treated with either phlorizin or MHY1485. **e** Cleaved caspase 3 positive cell number in the retinal GCL of the indicated groups of mice (n = 6/group). **f** Total cell number and NeuN positive cell number (normalized to the Hoechst nuclear counterstain) in the retinal GCL of the indicated groups of mice (n = 6/group). **g** Immuno-co-staining for pmTOR S2448 (green) and GFAP (red) of the retina from normal and 3-months diabetic mice treated with either phlorizin or MHY1485. Nuclei were counter-stained with Hoechst 33,342 (blue). GCL, ganglion cell layer; INL, inner nuclear layer; ONL, outer nuclear layer; *p ≤ 0.05; Scale bar: 50 µm

pmTOR S2448 and pS6 expression was decreased at 3 month of diabetes and PHL treatment recovered mTOR activity in the neuroretina as demonstrated by Western Blot and immunofluorescence assay (Fig. 8a, g). GLUT1 expression in the neuroretina of 3 m STZ-mouse was also increased following PHL treatment (Fig. 8a). MHY1485 treatment successfully activated mTOR in the neuroretina of 3 m STZ-mouse, since pmTOR S2448 level detected by WB and immunofluorescence assay, was high.

Beclin1 immunoreactivity in the retina from 3 m DM/PHL group resembled the similar expression pattern as those of normal control group: immunofluorescence signal was detected in the GCL, INL, and OPL. However, MHY1485 treatment resulted in stronger inhibition of autophagy, since Beclin1 signal was observed only in the GCL (Fig. 8b). Calbindin staining did not reveal any difference in OPL of the retina from all groups; meanwhile, the GCL differed between all experimental groups (Fig. 8b, c).

To assess, how autophagy affects the neuronal cells in the GCL, we immunostained the retinal tissues from 3mDM, 3mDM/PHL and 3mDM/MHY groups for Cleaved caspase 3 and NeuN, retinal tissues from normal mouse served as control. We observed a clear increase in Cleaved caspase 3 labeling in the GCL of 3 m STZ mouse, indicating high apoptotic activity after long-term hyperglycemia (Fig. 8d). PHL treatment resulted in moderate decrease of immunoreactivity and number of Cleaved caspase 3 positive cells (Fig. 8d, e). However, autophagy blocking with mTOR activator resulted in significant decrease of immunoreactivity and number of Cleaved caspase 3 positive cells comparing to those of 3 m DM and 3 m DM/PHL groups (Fig. 8d, e). Quantification of neuronal cells in the GCL of tissues from treatment groups also confirmed the neuronal cells rescue from apoptotic activity induced by long-term hyperglycemia (Fig. 8f). The total number of cells in GCL was decreased in the retinal tissues from 3mDM group comparing to those of from the normal group and 3mDM/PHL demonstrated a similar amount of Hoechst positive cells (*p* > 0.05). However, NeuN positive cell number was higher in tissues from 3mDM/PHL group comparing to 3mDM group (*p* > 0.05). In the tissues of the 3mDM/MHY group, total number of cells and NeuN positive cell was the highest among STZ-injected groups (*p* < 0.05) (Fig. 8f).

To confirm the neuronal rescue following autophagy modulation, we studied the glial response by GFAP labeling (Fig. 8g)*.* GFAP positive astrocytes and Müller glia resembling cells were detected in the NFL, GCL, and IPL of 3 m STZ-mouse. MHY1485 treated mouse retina demonstrated GFAP signals only in the NFL and GCL as demonstrated normal mouse retina. Similar results were found in those of PHL-treated mice (Fig. 8g).

## Discussion

The major contributor to the development of DR is the high levels of glucose in the blood [1, 50]. Glucose—the primary energy source of the cell—is a polarized large molecule, which cannot traverse the cell membrane by diffusion. In the retina, the proteins of facilitated diffusion glucose transporter family are proposed to be responsible for glucose transportation [51]. Among others, glucose transporter 1 (GLUT1) is the most studied and considered as the major transporter in the neural retina and blood-retinal barrier (BRB) [51–56]. As being insulin-independent glucose transporter, the GLUT1 expression and transport activity can be stimulated by the levels of glucose in the blood [57, 58]. Under diabetic conditions, hyperglycemia results in an increase of intracellular glucose concentration in the neural retina potentially through hyperglycemia-induced GLUT1 upregulation as an adaptive response [59–61]. However, long-term hyperglycemia may lead to the downregulation of GLUT1 in the retina [62–64]. Differential expression of GLUT1 depending on clinical stages of DR was also observed in postmortem human retina [52, 65, 66]. In the inner BRB, focal upregulation of GLUT1 expression was observed in long-standing DM patients with minimal or no clinical retinopathy [65]. However, GLUT1 expression was significantly decreased in human eyes with clinically evident DR [66]. Another study reported that GLUT1 immunoreactivity was not even observed in the neovascular tissue of patients with proliferative DR, which is an advanced stage [52]. Several mechanisms of GLUT1 downregulation are implicated upon long-term hyperglycemia including oxidative stress signaling and accumulation of advanced glycation end-products (AGEs) and their precursors, such as methylglyoxal, which, inter alia, may inhibit expression of the glucose transporter genes [67–70]. Also, growing data suggests another detrimental effect of long-term hyperglycemia on the retina which is manifested in the generation of autophagy processes through nutrient stress, oxidative stress, endoplasmic reticulum stress, hypoxia, inflammation, and others [39, 41, 71]. At this point, we hypothesized that the mTOR signaling pathway may be the link between the factors related to hyperglycemia and autophagy, consequently leading to the neuronal cell loss in DR (Fig. 1).

mTOR is atypical serine/threonine protein kinase from the phosphoinositide 3-kinase (PI3K)-related kinase family, which maintains the balance between anabolic and catabolic processes in response to a variety of physiological and pathological conditions [72]. mTOR pathway regulates myriad biological processes such as protein synthesis, proliferation, autophagy, metabolism, cell survival, and others through distinct protein complexes including mTOR complex 1 (mTORC1), mTOR complex 2 (mTORC2) and mTOR complex 3 (mTORC3) [72, 73]. The most intensively studied of all mTOR complexes is undoubtedly mTORC1. mTORC1 senses the main intracellular and extracellular signals, which are nutrient levels, growth factors, oxygen, amino acids, and stress, to regulate variety of essential metabolic processes, such as protein synthesis and autophagy [72].

High levels of nutrients, such as intracellular hyperglycemia directly or indirectly activates mTORC1 resulting in increased anabolism [72, 74]. In vitro experiments demonstrated that increased extracellular glucose stimulates GLUT1 expression and mTORC1 pathway, moreover, adenoviral overexpression of GLUT1 had similar effect on the latter [75]. Another study demonstrated that knockdown or inhibition of GLUT1 decreases retinal glucose, superoxide radicals and vascular endothelial growth factor in the early period of diabetes [76]. Activation of mTORC1 pathway in the early period of diabetes (28 days) has previously been reported in the retina of STZ-induced rat model [77]. In our study, 6 months monitoring of STZ-diabetic mice revealed biphasic expression of mTORC1 pathway proteins, demonstrating upregulation at 1 month and decreased expression in the following period (Fig. 3a, b). Of note, the upregulation of mTOR activity followed by downregulation has been reported in the other tissues of STZ-induced diabetes models, but the follow-up period lasted only 6 weeks [78]. The biphasic pattern was also observed in GLUT1 expression and activation of mTORC1 pathway correlated with the time point of enhanced expression of GLUT1 (Fig. 3a, b). Thus, activation of the mTOR pathway in the retina of STZ-induced diabetic mice is likely to be in response to hyperglycemia-induced GLUT1 upregulation leading to the increased glucose uptake. These findings have been confirmed by further experiments, where insulin independent glycemic control via PHL treatment resulted in downregulation of mTORC1, pS6 and GLUT1 proteins (Fig. 7a, b). PHL, a natural metabolite isolated from the plants, improves the hyperglycemia by preventing renal glucose resorption and intestinal glucose absorption through suppression of the sodium–glucose transporters [43]. Previous studies demonstrated the protective effect of PHL on the retina from diabetes by decreasing of blood glucose level [79, 80], which was also observed in our study (Fig. 2, Table1).

There is no consensus regarding the vascular or neural damage is the earliest morphological consequence of hyperglycemia in the diabetic retina. Nevertheless, the “feed-forward” concept suggests that neuronal dysfunction eventually compromises the overall integrity of the NVU, leading to neurodegeneration and vascular damage [33, 81]. Neurodegeneration is characterized by a decreased thickness of retinal nerve fiber layer, loss of ganglion cells and different types of amacrine cells [19, 20, 24, 26]. Neuronal cell loss was observed as early as after 1 month of STZ-induced diabetes [26]. Apoptosis is proposed to be the main degenerative mechanism in DR [34]. Therefore, we evaluated the immunoreactivity of cleaved caspase 3 in the diabetic retina, which was gradually increased in the GCL over the follow-up period in line with the results of other studies (Fig. 3c, d) [27]. The reduction of NeuN immunoreactivity and the total number of cells in the GCL also indicated hyperglycemia-induced neuronal cell damage (Fig. 3c, e), mirroring the results of previous studies [26]. We assume that there may be a functional relationship between the expression of ribosomal protein S6 in the GCL and reduced immunoreactivity of NeuN in the diabetic retina (Fig. 4f), since pS6 is one of the downstream effector of mTORC1 pathway on protein synthesis and the part of translational machinery [82]. Further studies are needed to evaluate the significance of neuronal expression of pS6 in the context of neurodegeneration in DR.

**Fig. 9:**
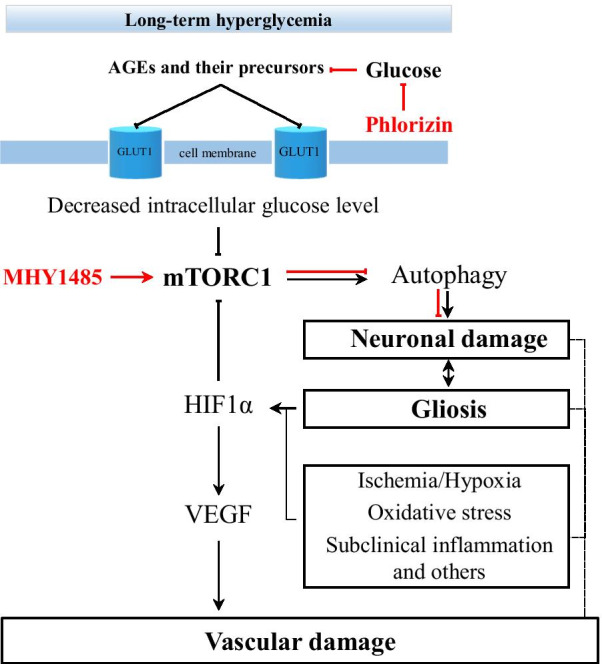
Schematic representation of the mechanism of neuronal cell rescue by autophagy modulation

One chain of pathological processes triggered by hyperglycemia-related conditions is autophagy, which may cause neurodegeneration in DR [41]. In vitro experiments demonstrated that autophagy strongly depends on the deactivation of the mTORC1 pathway which could be caused by various factors including energy deprivation and hypoxia in terms of the pathogenesis of DR [41, 71, 83]. Inhibition of the mTORC1 pathway initiates the cascades of events leading to the increased expression of autophagic proteins, including LC3B and Beclin1 [71]. Beclin1 is recruited during the early phases of degradative autophagy flux and its upregulation was found to contribute to the initial deregulation of autophagy in human diabetic retinas as well as in STZ-induced diabetes models [36, 38, 84]. To the best of our knowledge, there currently exists no report regarding the involvement of the mTOR pathway in autophagy-induced ganglion cell loss in DR (Additional file 1: Fig. S1). In our study, upregulation of Beclin1 in the entire inner retina was accompanied with the signs of neuronal cell damage, such as activation of apoptotic marker—cleaved caspase 3 and decrease of the total number of cells in the GCL, along with depleted NeuN immunoreactivity in retinal ganglion cells (Figs. 6b and 8c–g). Furthermore, autophagy deregulation was also confirmed by upregulated ATG9A in NeuN positive RGCs of 3 m DM mouse retina (Additional file 1: Fig. S2). PHL treatment confirmed that hyperglycemia-induced autophagy deregulation is responsible for neuronal cell loss and mTORC1 pathway activity is required to preserve neurons in DR (Fig. 8). The number of apoptotic cells was decreased and NeuN immunoreactivity was higher in the GCL of PHL-treated mice retina comparing to that of 3 m diabetic control mice (Fig. 8c–g). However, blockade of autophagy by mTOR activator—MHY1485 injections resulted in the more prominent rescue of neuronal cells, as apoptotic cell number in the GCL was the lowest among retinas of all diabetic groups (Fig. 8e). In addition, the total number of cells as well as NeuN positive cells in MHY1485 treated retina was higher than retinas from other diabetic groups (Fig. 8f, g). The less beneficial effect of glycemic control on the ganglion cells comparing to autophagy blockade is likely due to cumulatively inhibition of the mTORC1 pathway by hyperglycemia-related conditions, consequently results in autophagy dysregulation in the diabetic retina. As a result, glycemic control could not fully resolve ganglion cell death in DR (Fig. 8d–f). Moreover, the fact that glycemic control could not restore mTORC1 pathway activity, as we observed in the MHY1485 treated mouse retina, supports this assumption (Fig. 8a). These findings suggest that mTOR pathway plays a crucial role in retinal ganglion cell damage promoted by diabetes and modulation of mTORC1 activity has the rescue effect on the retina through inhibition of autophagy even in uncontrolled hyperglycemia conditions (Fig. 9).

## Conclusion

Our study suggests that during the early period of diabetes, hyperglycemia-induced GLUT1 activation results in increased glucose uptake and mTORC1 signaling activity. However, long-term hyperglycemia-related conditions cause mTOR inhibition leading to autophagy dysregulation, hence the reason for the neuronal cell death in DR. Understanding the mechanism of apoptosis in retinal ganglion cells in the pathogenesis of diabetic retinopathy may reveal novel approaches to prevent the development of the disease at early stages. Also, this study provides the first experimental evidence for the efficacy of mTOR activator—MHY1485 in STZ-induced diabetic retinopathy. Thus, the current study not only describes the mechanisms of neurodegeneration through hyperglycemia-/mTOR/autophagy/apoptosis pathway, but also provides a rationale for the development of new strategies based on autophagy modulation to manage neuronal cell death in the early period of DR.

## Supplementary Information

**Additional file 1: Supplemental Table S1**. Details on antibodies. **Supplemental Figure S1**. The potential interactions among selected proteins. Network nodes represent proteins (Slc2a1 (GLUT1), Mtor, Rps6, Becn1, Hif1a, Vegfa, GFAP, Casp3, RBFOX3 (NeuN)) of Homo sapiens and Mus musculus. Splice isoforms or posttranslational modifications are collapsed, i.e. each node represents all the proteins produced by a single, protein-coding gene locus. Legends: Node Color: colored nodes: query proteins and first shell of interactors; white nodes: the second shell of interactors Node Content: empty nodes: proteins of unknown 3D structure; filled nodes: some 3D structure is known or predicted Edges represent protein-protein associations. Meaning of network edges: evidence – (a) and molecular action (b). **Supplemental Figure S2**. Autophagy activation in retinal ganglion cells Immuno-co-staining for ATG9A (green) and NeuN (red) of normal and 3 m DM mouse retina.

## Data Availability

The datasets used and/or analyzed during the current study are available from the corresponding author on reasonable request.

## Abbreviations

AGEs: Advanced glycation end-products
BRB: Blood-retinal barrier
DM: Diabetes mellitus
DR: Diabetic retinopathy
GCL: Ganglion cell layer
GFAP: Glial fibrillary acidic protein
GLUT1: Glucose transporter 1
GS: Glutamine synthetase
INL: Inner nuclear layer
IPL: Inner plexiform layer
mTOR: Mechanistic target of rapamycin
NFL: Nerve fiber layer
NVU: Neurovascular unit
ONL: Outer nuclear layer
PB: Phosphate buffer
PBS: Phosphate-Buffered Saline
PFA: Paraformaldehyde
PHL: Phlorizin
