# Supplementary material for: mTOR-dependent dysregulation of autophagy contributes to the retinal ganglion cell loss in streptozotocin-induced diabetic retinopathy
Source: Cell Commun Signal. 2021 Feb 26;19:29. doi: 10.1186/s12964-020-00698-4 (PMC7913405; doi:10.1186/s12964-020-00698-4)
Supplement: Supplementary file 2 — Additional file 1: Supplemental Table S1. Details on antibodies. Supplemental Figure S1. The potential interactions among selected proteins. Network nodes represent proteins (Slc2a1 (GLUT1), Mtor, Rps6, Becn1, Hif1a, Vegfa, GFAP, Casp3, RBFOX3 (NeuN)) of Homo sapiens and Mus musculus. Splice isoforms or posttranslational modifications are collapsed, i.e. each node represents all the proteins produced by a single, protein-coding gene locus. Legends: Node Color: colored nodes: query proteins and first shell of interactors; white nodes: the second shell of interactors Node Content: empty nodes: proteins of unknown 3D structure; filled nodes: some 3D structure is known or predicted Edges represent protein-protein associations. Meaning of network edges: evidence – (a) and molecular action (b). Supplemental Figure S2. Autophagy activation in retinal ganglion cells Immuno-co-staining for ATG9A (green) and NeuN (red) of normal and 3 m DM mouse retina. [file 12964_2020_698_MOESM2_ESM.pdf]

#	Antibody	Company	Cat no.	Application	Dilution
**1**	phospho-mTOR (Ser2448)	Abcam	ab109268	WB<br>IF	1:1000<br>1:500
**2**	mTOR	Cell Signaling Technology	#2983	WB	1:1000
**3**	phospho-S6 Ribosomal Protein (Ser240/244)	Cell Signaling Technology	#5364	WB	1:1000
**4**	GLUT1	Abcam	ab 40084	WB<br>IF	1:1000
			ab115730	IF	1:1000
**5**	β-actin	Santa Cruz Biotechnology	sc-47778	WB	1:1000
**6**	Brn3a	Millipore	MAB1585	IF	1:100
**7**	NeuN	Millipore	MAB377	IF	1:2000
**8**	Calbindin	Sigma Aldrich, Inc.	C9848	IF	1:100
**9**	Glutamine synthetase	Millipore	MAB302	IF	1:1000
**10**	Cleaved caspase 3	Cell Signaling Technology	CST9661	IF	1:400
**11**	Beclin1	Novus Biologicals	NB-500-249	IF	1:1000
**12**	GFAP	Abcam	ab53544	IF	1:300
**13**	ATG9A	Novus Biologicals	NB110-56893	IF	1:1000

Supplemental Table S1. Details on antibodies

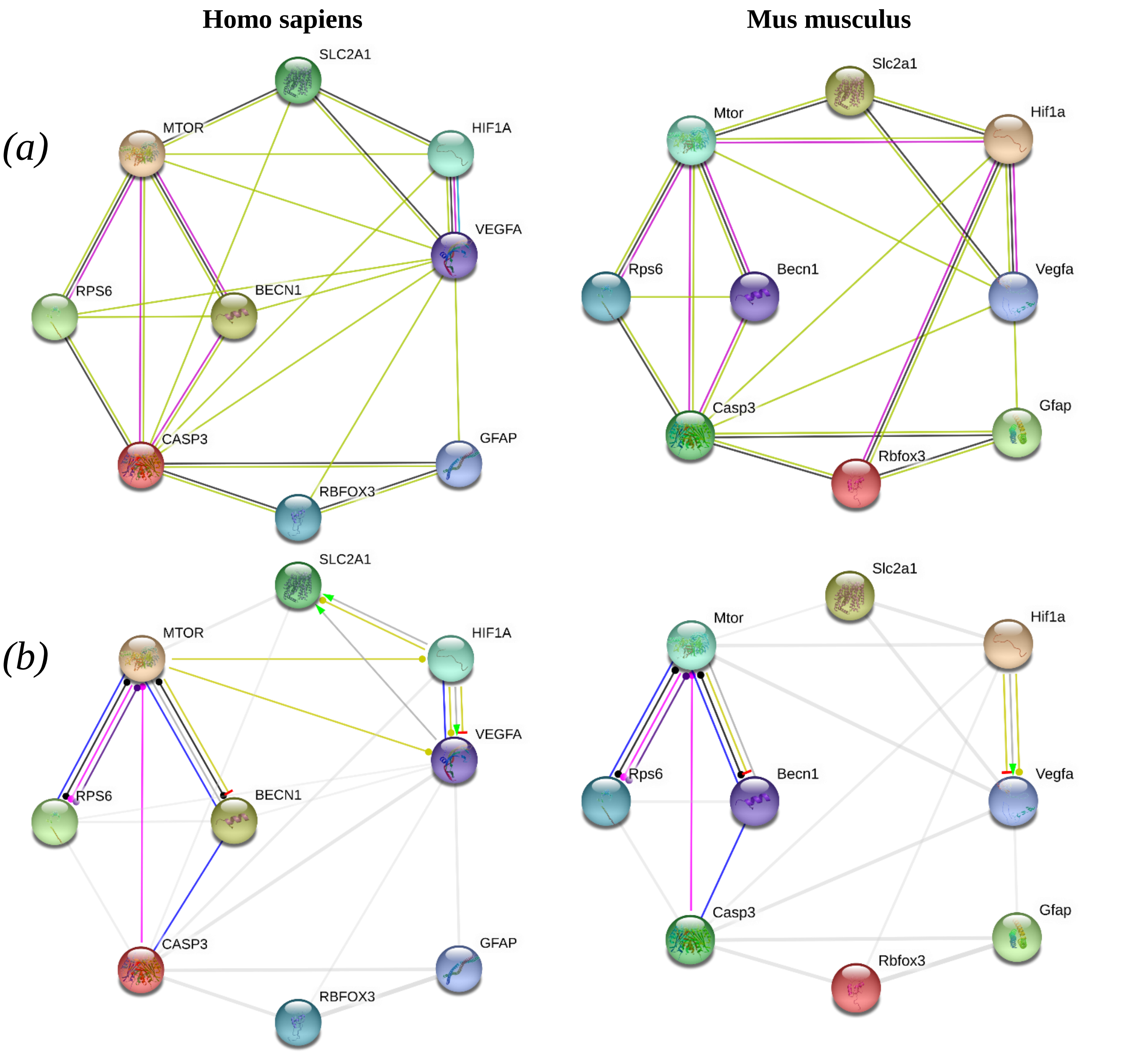

Supplemental Figure S1. The potential interactions among selected proteins.

Network nodes represent proteins (Slc2a1 (GLUT1), Mtor, Rps6, Becn1, Hif1a, Vegfa, GFAP, Casp3, RBFOX3 (NeuN)) of Homo sapiens and Mus musculus. *Splice isoforms or post-translational modifications are collapsed, i.e. each node represents all the proteins produced by a single, protein-coding gene locus.*

Legends:

Node Color: *colored nodes: query proteins and first shell of interactors; white nodes: second shell of interactors*

Node Content: *empty nodes: proteins of unknown 3D structure; filled nodes: some 3D structure is known or predicted*

Edges represent protein protein associations. Meaning of network edges: evidence – *(a) and* molecular action *(b)*

Known interactions:
- from curated databases
- experimentally determined

Predicted interactions:
- gene neighborhood
- gene fusions
- gene co-occurrence

Others
- textmining
- co-expression
- protein homology

Action types
- activation
- binding
- phenotype
- reaction
- inhibition
- catalysis
- posttranslational modification
- transcriptional regulation

Action effects:
- positive
- negative
- unspecified

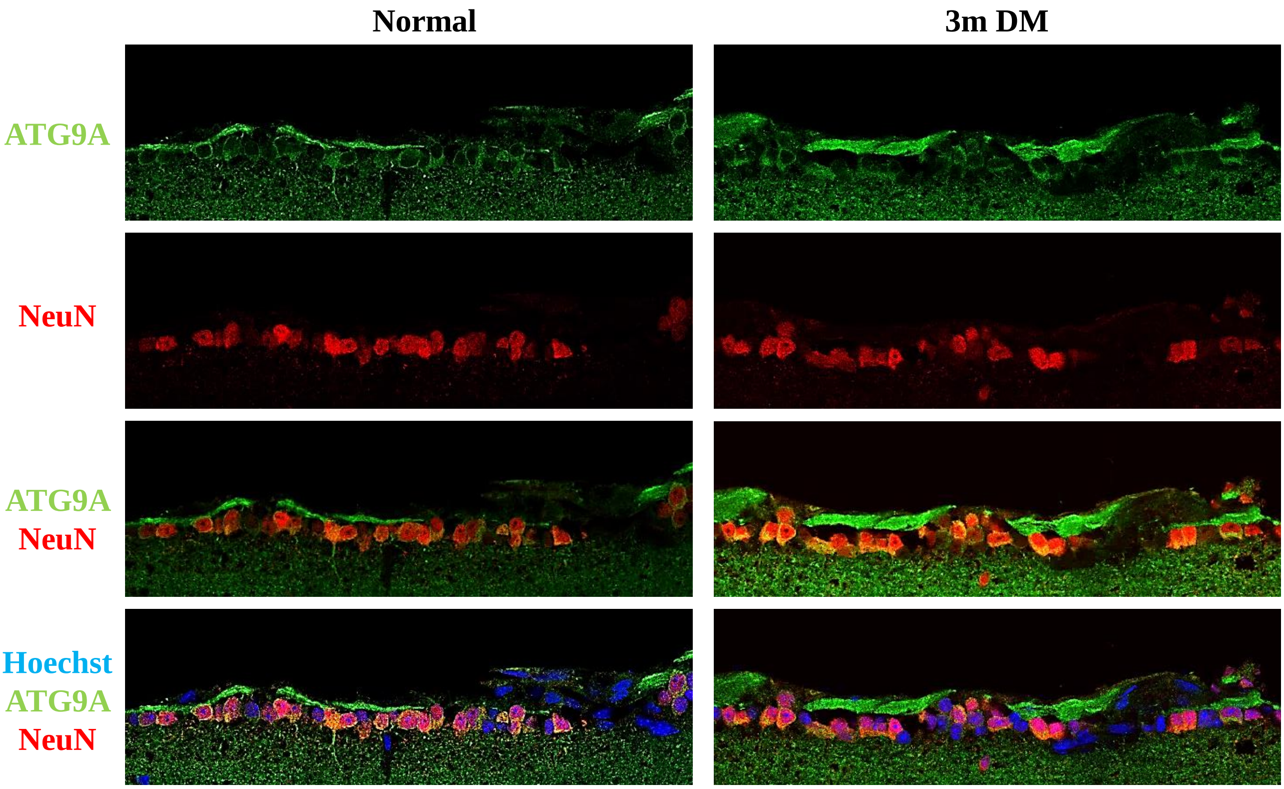

Supplemental Figure S2. Autophagy activation in retinal ganglion cells.
Immuno-co-staining for ATG9A (green) and *NeuN* (red) of normal and 3 m DM mouse retina.
